# Data‐Driven Implementation Trials: Realizing Their Full Potential in Achieving the Promise of Learning Health Systems

**DOI:** 10.1002/lrh2.70043

**Published:** 2025-10-19

**Authors:** Charis X. Xie, Patricia D. Franklin, Theresa L. Walunas, Rinad S. Beidas

**Affiliations:** ^1^ Wolfson Institute of Population Health Queen Mary University of London London UK; ^2^ Department of Medical Social Sciences, Feinberg School of Medicine Northwestern University Chicago Illinois USA; ^3^ Division of General Internal Medicine, Department of Medicine, Feinberg School of Medicine Northwestern University Chicago Illinois USA

**Keywords:** implementation trials, learning health systems, routine healthcare data

## Abstract

The digital transformation of healthcare has generated unprecedented volumes of routine clinical data, enabling health system leaders, including quality improvement (QI) efforts, to optimize care using real‐time analytics. However, health system QI typically focuses on changes within localized environments; it is often limited in its ability to address systemic barriers or scale evidence‐based strategies across diverse settings. Thoughtful integration of implementation science (IS) approaches addresses this gap by systematically integrating interventions into diverse practice settings and defining generalizable implementation strategies. These attributes position IS as a cornerstone of learning health systems (LHS), which strive for population‐wide improvements through continuous, data‐driven learning. Within this paradigm, randomized implementation trials provide the gold standard for comparing and optimizing implementation strategies. By leveraging routine data, these trials generate causal evidence on the effectiveness of different approaches and offer rigorous insights for health system decision‐makers. In this viewpoint, we highlight data‐driven implementation trials as catalysts for rigorous and scalable health system transformation. Specifically, we articulate the value proposition of data‐driven implementation trials, examine their transformative potential toward learning health systems, and outline persistent challenges. Drawing on experiences from the UK and the US in large health systems, we propose actionable recommendations to optimize infrastructure, foster collaboration, secure health system‐level commitments, and cultivate a culture that is grounded in IS while augmenting the impact of QI—critical steps toward realizing scalable, equitable healthcare innovation.

## 1 Background

The widespread adoption of health information technologies, particularly the electronic health record (EHR), has generated unprecedented volumes of healthcare data. This digital transformation has enabled sophisticated data‐driven approaches to inform clinical decision‐making, improve patient outcomes within healthcare settings, and accelerate progress toward continuous learning health systems (LHS)—systems that align science, informatics, incentives, and culture to continuously improve care, engage patients, embed best practices, and generate new knowledge [1]. While data and analytic methods have evolved rapidly, today's health system practice transformation methods are generally defined by traditional quality improvement (QI) programs. Although QI methods have been embedded in healthcare systems for decades, the recent LHS goal to accelerate widespread adoption of best clinical practices challenges quality programs to go beyond local project‐based improvements and address multi‐level factors and strengthen the generalizability of practice change strategies across diverse healthcare settings [2]. The demands of the LHS call for broader approaches to optimally implement best clinical practices to improve health and health care.

Implementation science (IS) provides systematic frameworks and methods for incorporating evidence‐based interventions and data analytics into diverse healthcare systems and practices. This discipline examines barriers and facilitators to new practice implementation, informs and develops multi‐level strategies for successful integration, and evaluates how these strategies support clinician behavior change within organizational constraints [3]. Together with quality improvement programs, these characteristics position IS as a cornerstone of the learning health systems in achieving population health improvements [4]. Within this LHS paradigm, we emphasize the importance of translating novel data‐driven best practices into healthcare through randomized implementation trials (hereafter implementation trials). Unlike observational IS studies and local QI, these IS trials provide causal evidence about implementation strategies, generating rigorous and actionable insights to guide decision‐making. Table 1 summarizes key distinctions between QI projects, observational IS studies, and implementation trials. While fully leveraging routine clinical data, implementation trials do more than evaluate the strategies themselves. They also prototype the feedback loops and infrastructure the LHS requires, transforming isolated evidence into system‐wide learning [5].

**TABLE 1:** Differences between QI projects, observational IS studies, and implementation trials.

Feature	Traditional QI	Observational IS studies	Implementation trials
Primary goal	Local improvement in health service/care processes	Understand determinants and implementation processes	Test strategies for implementation, scale‐up, and sustainment; understand mechanisms
Focus	Specific issues within a single system	Patterns across multiple contexts or cases	System‐level strategies across multiple sites/contexts
Design	Iterative Plan‐Do‐Study‐Act cycles	Observational, mixed methods	Experimental, often (cluster) randomized allocation with control/comparison group
Generalizability	Results often not generalizable beyond site	Correlations and associations	Causal inference, with potential scalability across systems
Outputs	Local process metrics	Implementation determinants	Evidence on effective strategies for system‐wide implementation and scale‐up

Although the potential of such data‐driven implementation trials has been noted in the literature [5, 6, 7], their role remains underexplored and not yet fully realized. In this viewpoint, we highlight data‐driven implementation trials as catalysts for rigorous and scalable health system transformation. We first explore the value proposition of data‐driven implementation trials. We then draw on perspectives from both the UK and the US in large health systems and propose recommendations for enhancing the potential of these trials in achieving the promise of learning health systems.

## 2 Data‐Driven Implementation Trials

Implementation trials primarily evaluate the effects of implementation strategies (in contrast to clinical interventions) on implementation outcomes (e.g., adoption, fidelity, sustainability) to continuously improve healthcare delivery. In some cases, these trials use effectiveness–implementation hybrid approaches, allowing for a dual evaluation of both implementation strategies and clinical effectiveness [8]. In recent years, implementation trials have leveraged routinely collected health data—embedded in electronic health records, registries, and administrative datasets—to identify eligible participants and monitor patient outcomes, and deploy informatics tools to deliver interventions [5]. These routine data provide rich population health information, including demographic details, national reference indicators, and health event records [9] that possess great value for healthcare research. By minimizing the need for bespoke data collection, these data‐driven implementation trials may reduce costs, enhance efficiency, and improve generalizability to real‐world settings.

Data‐driven implementation trials move beyond the traditional ‘implement and hope’ approach [10], offering health systems a precision tool to reduce uncertainty, generate causal insights, and accelerate improvement. When embedded within care delivery, these trials create a dynamic learning system—enabling rapid‐cycle evaluation and system‐wide transformation [5, 11].

The primary value of this approach lies in its ability to reduce risk in major organizational implementation efforts and set them up for success given that the majority of implementation efforts end in failure [12]. By gathering actionable data about what works in what contexts, leaders can make informed decisions for adapting implementation strategies, which could reduce the financial and operational risks associated with implementing or scaling up novel best practice interventions. Historical data about implementation processes, outcomes, and challenges can help in designing more “resilient” strategies to integrate evidence into practice. For example, in our recent implementation trial of a secure firearm storage program in US pediatric primary care [13] (Box 1), we found that an EHR strategy combined with facilitation was more effective than an EHR nudge alone, with only modest incremental resource investment. This evidence equips health system leaders with data to prioritize resource allocation toward facilitation strategies, particularly when scaling interventions across diverse clinical settings. Importantly, we are now adapting and testing our most effective implementation approach in health settings that are less well resourced (i.e., federally qualified health centers which primarily serve low‐income, uninsured, and medically underserved populations), allowing for scale‐out to new settings.

BOX 1 **ASPIRE Secure Firearm Storage Trial.**

The Adolescent and Child Suicide Prevention in Routine Clinical Encounters (ASPIRE) trial tested strategies to increase delivery of an evidence‐based secure firearm storage program (SAFE Firearm) in pediatric primary care. SAFE Firearm consists of brief counseling with parents on secure firearm storage and provision of free cable locks during pediatric well‐child visits. In this unblinded cluster randomized trial, 30 clinics across two large US health systems in Michigan and Colorado were randomized to receive either [1] an electronic health record (EHR) documentation template embedded in the standard well‐child visit workflow (nudge), or [2] the EHR template plus clinic‐level facilitation support (nudge+).

Outcomes were extracted from the EHR for all well‐child visits (*n* = 47,307) among children aged 5–17 years over 1 year. The primary outcome—delivery of both counseling and a lock, as documented in the EHR —occurred in 49% of visits in the nudge+ arm compared to 22% in the nudge arm. The study demonstrates how the EHR can prompt intervention delivery and facilitate outcome analysis, enabling scalable evaluation across large, diverse populations.

Second, data‐driven implementation trials could accelerate return on investment (ROI) by allowing health systems to detect ineffective strategies early and avoid wasted resources.

Real‐time data analysis using digital dashboards enables health systems to rapidly identify successful implementation components and abandon those that don't work, thereby optimizing resource allocation and reducing waste. For instance, when implementation barriers are detected early through continuous monitoring of clinician adoption, adjustments can be made before vast amounts of money are expended on ineffective strategies. A practical example is the series of rapid‐cycle, randomized controlled trials embedded within quality improvement projects at NYU Langone Health [14]. In these projects, iterative testing and real‐time data feedback enabled the institution to quickly identify and discontinue ineffective interventions, replacing them with more successful alternatives, resulting in immediate cost efficiencies [10]. These studies demonstrate how embedding randomization directly into routine workflows and leveraging EHR data for outcomes can provide a rigorous yet agile method for evaluating implementation strategies, establishing a sustainable infrastructure for rapid, data‐driven learning. Box 2 summarizes two illustrative trials from this program, focused on optimizing clinical decision support alerts through EHR‐embedded A/B testing [15]. When scaled up, such trials could identify generalizable implementation strategies, broaden health outcome improvement, and sustain ROI gains across diverse settings.

BOX 2 **Rapid A/B Testing of Clinical Decision Support Alerts.**

NYU Langone Health developed an EHR‐embedded framework to conduct rapid‐cycle randomized controlled trials of clinical decision support (CDS) alerts, with the goal of reducing alert fatigue while maintaining effectiveness.

The first trial focused on inpatient influenza vaccination. Adults eligible for vaccination were identified and randomized through EHR data, the system randomized patients into two arms: the existing alert and a revised alert that varied in placement and visual presentation. Outcomes included vaccination orders placed (clinician acceptance) and the number of alerts displayed per patient per day (a proxy for alert fatigue). The revised alert text alone produced minimal change in alert frequency, but this null result prompted further technical refinements (removing the dismissal button and suppressing alerts in procedural areas), which ultimately reduced alerts dramatically (from 23.1 to 7.3 per patient per day) without lowering vaccination order rates.

The second experiment focused on outpatient tobacco cessation alerts. Smokers identified in the EHR triggered clinician‐facing alerts, which were randomized at the ambulatory practice level to display different message framings—financial (highlighting potential reimbursement), evidence‐based (emphasizing quality of care), or regulatory (underscoring institutional expectations)—with complementary images added in later rounds. Across three A/B testing experiments conducted over 8 months, no significant differences were observed in clinician acceptance rates, measured by documentation of counseling and related orders. These null results highlighted the potential value of rapid‐cycle testing in identifying ineffective strategies early, thereby enabling health systems to redirect resources more efficiently.

Third, one can imagine multicenter health systems and/or state‐wide practice networks adopting IS methods to support scalability and generalization by efficiently implementing and testing implementation strategies for embedding new best practices into routine care. Data‐driven implementation trials, focused on optimizing the process of integrating evidence into practice, represent a methodologically rigorous approach toward establishing effective learning health systems. By embedding implementation trials within routine health system operations, routine data becomes a source of actionable insights, driving a loop of strategy testing, adjustment, and optimization, creating rapid learning cycles that accelerate the adoption of evidence‐based practices in healthcare settings [5]. For example, trials might compare strategies like clinician training programs, audit‐and‐feedback systems, or tailored stakeholder engagement to identify the most effective ways to adopt guidelines‐ as exemplified by a nationwide implementation trial (IMP^2^ART) [16] in UK primary care, which uses routine data to evaluate a whole‐system strategy for supported asthma self‐management across England and Scotland (Box 3). Over time, these feedback cycles allow health systems to accumulate valuable data and experience, deepening their understanding of local contexts and capabilities. Successful implementation strategies can then be translated from one clinical condition to another with contextual refinement to support maintenance. Consequently, future implementations may be more efficient, as teams can build on previously successful strategies and avoid known pitfalls. Critically, this approach shifts the focus from whether an intervention works to how systems can reliably implement what works, accelerating the translation of evidence into sustained practice.

BOX 3 **Implementing Improved Asthma Self‐Management as Routine (IMP^2^ART) Trial.**

IMP^2^ART is a UK‐wide cluster randomized hybrid II implementation trial designed to embed supported asthma self‐management in routine primary care. Supported self‐management—centered on a personalized written asthma action plan reinforced by regular professional review—has a strong evidence base, reducing unscheduled consultations and improving asthma control and quality of life. IMP^2^ART evaluates whether a structured, system‐level implementation strategy can increase adoption of this approach.

A total of 144 general practices across England and Scotland were randomized to either the implementation strategies or usual care. The strategies combine professional training, patient‐facing resources, and organizational supports, including audit and feedback and an asthma review template within the EHR. The primary outcomes are unscheduled care from the EHR, and ownership of asthma action plans reported by patients identified and mailed via the EHR. Routine data are used throughout the trial to assess clinical outcomes, support questionnaire mailings, conduct health economic analyses, and deliver intervention components such as audit and feedback. Although trial results have not yet been published, IMP^2^ART illustrates a model of how large‐scale, data‐driven implementation trial can pragmatically test strategies across hundreds of practices, generating evidence to inform national policy for long‐term condition management.

Fourth, data‐driven implementation trials can advance health equity in two ways: through the use of population‐level data and through equity‐oriented trial design. Routine health data provide rich information that can be leveraged to identify disparities and uncover their root causes [17]. For example in England, researchers developed a set of health equity indicators for the National Health Service (NHS) using routine data from general practices, hospital records, and vital statistics. These indicators quantified gaps between the most and least deprived communities across measures such as primary care supply, chronic disease hospitalizations, and amenable mortality, thereby signaling where equity‐focused quality improvements were most urgently needed [18]. Data‐driven implementation trials can then build on these insights by embedding equity throughout the research process—posing equity‐focused questions [19], selecting and tailoring implementation strategies to address disparities [20] and measuring outcomes such as reach, representativeness, and differential effects across groups [21]. Together, these approaches ensure that implementation trials do not merely generate evidence but actively contribute to narrowing health inequities.

Despite the promises, there are important pitfalls to consider. A first set of challenges is technical. Data quality issues such as accuracy, completeness, and granularity are long‐standing challenges in routine data research and are even more significant issues when relying on EHR data, which derives from immediate clinical care needs as well as billing requirements. Inconsistent coding, missingness, and limited standardization in EHR documentation can undermine trial validity. Addressing these issues often requires extensive investment in infrastructure and dedicated data quality initiatives. System‐specific barriers also limit the scalability of data‐driven implementation trials. In the UK, the legacy EHR systems have hindered the inclusion of certain practices due to compatibility issues [22], while in the US, the general lack of interoperability across EHR systems may necessitate duplicative programming of implementation strategies.

Another set of pitfalls concerns the complexity of measurement. Implementation outcomes are inherently difficult to define and capture. Clinician adoption (the uptake of the intervention) and fidelity (the extent to which the intervention is delivered as intended) are commonly measured by routine data in implementation trials [5]; however, other implementation outcomes, such as penetration (the extent to which the intervention is integrated across eligible settings) and sustainability (the continued use of the intervention over time), can be difficult to measure, and some indicators may not be routinely captured in available data sources. For example, while the IMP^2^ART trial (Box 3) leveraged EHR data to capture a nationwide population, evaluate strategies, and conduct both process and health economic evaluations, some key implementation outcomes—such as patient ownership of asthma action plans—were not routinely collected across all trial arms [16].

A further set of barriers relates to the substantial resource and workforce demands for adopting implementation trials—including educating clinicians and staff about trial methodologies, securing their acceptance, and providing ongoing training to support implementation. Without leadership buy‐in and integration into existing workflows, even well‐designed interventions may fail to be delivered as intended. At the policy level, regulatory frameworks can sometimes impede the efficient use of routine data in implementation trials. For example, in the UK, while the Health Research Authority (HRA) provides central approval, additional site‐specific approvals may still be required from individual NHS Trusts or Health Boards, particularly when local capacity and capability checks or non‐NHS sites are involved. These organizational and policy factors are often underestimated but are central to implementation success.

## 3 Call to Action

To unlock the full potential of data‐driven implementation trials to accelerate the realization of the learning health system, a committed and collaborative approach is essential. Healthcare system leaders, policymakers, and funders need to raise awareness, prioritize sustainable funding, invest in robust data infrastructure, ensure that IS experts are working alongside quality improvement teams, implement supportive policies, and foster partnerships. Table 2 summarizes these recommendations at a glance. While a fully mature learning health system is a long‐term goal, the following interim steps can begin generating value immediately and set the stage for that broader vision.

**TABLE 2:** Recommendations to advance data‐driven implementation trials.

Priority areas	Actionable recommendations	Actors involved
Secure whole system‐level support	Allocate dedicated funds to embed implementation trials within learning health system initiatives Allocate resources that encourage routine data collection and analysis as well as set strategic priorities that incorporate implementation activities into everyday practice Integrate implementation scientists into routine workforce structures and provide accessible training for practitioners	Health system leaders, funders, policymakers
Optimize the infrastructure	Invest in robust data infrastructure by improving clinical documentation at the source Advance interoperability through standardized data formats Common data models and APIs Develop flexible platforms that integrate diverse data types (e.g., EHRs, registries, genomics, patient‐reported outcomes, social determinants) Build standardized and reusable infrastructures that support multi‐site collaboration and allow trials to continuously leverage shared data resources	Health IT developers, CIOs, national digital health agencies
Foster collaboration and partnership	Forge public–private partnerships to align incentives and resources to support multi‐site implementation trials Promote international partnerships that pool expertise and infrastructure to develop and adapt implementation strategies across diverse contexts Engage clinicians, patients, and communities as co‐producers to strengthen design, recruitment, and relevance while fostering trust and ownership	Research consortia, patient advocacy groups, health systems
Cultivate a new culture	Cultivate a culture that values integration of IS and QI for systematic, scalable learning Incorporate IS methods and implementation trials into medical, QI, and workforce development curricula	Academic institutions, professional societies, health systems

### 3.1 Secure Whole System‐Level Support

Data‐driven implementation trials to serve the LHS vision will need buy‐in from all parts of the healthcare ecosystem to be sustainable and impactful. From the top down, healthcare funders and policymakers can allocate dedicated funds to build a supportive environment for implementation studies. This funding should support not only the trials themselves but also the associated infrastructure and workforce training needed to make these trials a norm. Emphasizing trial efficiency, equity, long‐term cost‐effectiveness, and multi‐system patient benefits can strengthen the case for this investment. A useful precedent is the US Agency for Healthcare Research and Quality's EvidenceNOW initiative, a $112 million national program designed to strengthen primary care by helping practices apply research evidence in routine care [23]. Through seven regional cooperatives, it supported more than 1700 small and medium‐sized practices by providing external facilitators, IT and EHR assistance, and learning networks, and demonstrated modest but meaningful improvements in clinical outcomes as well as in practices' capacity for ongoing quality improvement [24, 25]. EvidenceNOW illustrates how targeted, system‐level investment in infrastructure and workforce can enable practices to adopt evidence more effectively—offering a model for how implementation trials could be supported at scale.

In parallel, it is also important for health system leadership to allocate resources and establish policies that encourage routine data collection and analysis, as well as set strategic priorities that incorporate implementation activities into everyday practice. Cultivating such an organizational culture enables data‐informed decision‐making based on rigorous methods and contributes to the value of continuous improvement. For example, the UK's Building a Digital Ready Workforce Program strengthens the NHS's data‐driven culture by investing in leadership development, professionalizing informatics roles through the Faculty of Clinical Informatics, enhancing digital literacy across staff, and running campaigns to attract digital talent [26].

From the bottom up, workforce and practitioner training are needed to increase awareness and interest in conducting data‐enabled implementation trials. Training should prioritize existing QI teams, building their skills in data collection, analysis, and interpretation to work confidently and accurately with real‐world data and with multi‐institution initiatives. Implementation scientists should be a part of existing QI teams. For practitioners interested in expanding their knowledge of implementation science, foundational knowledge could be incorporated through optional, accessible resources like seminars or short training courses that complement their clinical roles. With this collaborative approach, practitioners can align their activities with an implementation plan from the outset, helping to proactively address some of the common implementation challenges that may arise. As an example, a Massachusetts state LHS collaborative is training embedded researchers on the principles of both QI and IS to serve future multi‐site collaborations [27].

### 3.2 Optimize the Infrastructure

For implementation trials to better use routine data, healthcare systems must invest in a robust data infrastructure that enables seamless collection, integration, and analysis. The first step is to improve data quality at the source by addressing clinical documentation challenges such as workload and administrative burden. Approaches include redesigning EHR interfaces with intuitive, user‐friendly, structured data entry forms and deploying natural language processing or AI tools that auto‐populate fields or recommend standardized codes [28, 29]. By improving documentation, health systems can create a cycle where better data benefits both clinicians and patients.

Next, infrastructure must enable interoperability that allows information sharing across multiple healthcare sites and systems using standardized data formats, common data models, and APIs. A prime example is KP HealthConnect, launched by Kaiser Permanente, which integrates patient information across all care settings, connects uniform billing and ancillary systems, and provides members with access to personal health records [30, 31]. This well‐established data infrastructure supports many of its trials in adopting an adaptive data processing approach, allowing preliminary analyses to effectively inform trial design [22].

Ideally, a flexible and scalable infrastructure can accommodate different data types and expand over time. Alongside commonly used data sources like EHR, registries, and administrative data, it is also essential to include new types of data such as wearable data, genomics, patient‐reported outcomes, social determinants of health, and so on. This will support a comprehensive view of implementation outcomes across diverse contexts and patient demographics. For example, the UK Biobank integrates electronic health records, lifestyle and imaging data, and genetic data into a single research platform [32]. Similarly, the All of Us Research Program in the US combines EHRs, survey data, genomic information, and wearable device data (e.g., Fitbit) to support research across diverse populations and health contexts [33]. In low‐resource settings, strategies should focus on cost‐effective solutions such as leveraging open‐source software, implementing basic EHRs, and utilizing mobile health technologies [34].

A consistent and reusable infrastructure is essential to build trial capacity that endures beyond individual trials [35]. Currently, many infrastructures are still developed on a trial‐by‐trial basis, resulting in resources such as research databases created for one study not being applicable to another. A standardized infrastructure would allow health systems to continuously utilize shared resources, facilitate multi‐site collaboration, and support the scalability of implementation studies, ultimately enhancing multicenter research capacity and accelerating the translation of findings into practice. A working example is the Patient‐Centered Outcomes Research Institute (PCORI) research infrastructure (PCORNet), which enables multiple pragmatic trials and observational studies to run on the same platform by continuously leveraging shared data resources and standardized tools [36].

### 3.3 Foster Collaboration and Partnership

Healthcare systems can cultivate partnerships that bring together the public and private sectors. In public health systems, public‐private partnerships enable governments to leverage private‐sector resources, technology, and expertise. For example, the UK NHS partners with tech firm Palantir to develop a Federated Data Platform, integrating real‐time data to help NHS staff optimize resource allocation (e.g., operating theater slots and outpatient clinic capacity) to reduce delays and improve patient care [37]. In private health systems, public‐private partnerships focus on scaling innovations or aligning corporate capabilities with public health priorities. The US federal government collaborated with pharmaceutical companies such as Pfizer, Moderna, and Johnson & Johnson to accelerate COVID‐19 vaccine development and distribution, combining federal funding, logistics, and regulatory support with private‐sector R&D and manufacturing [38]. In another example, Blue Cross Blue Shield (BCBS) of Michigan has funded successful QI collaboratives to support health systems across the state to generate uniform data and improve health outcomes [39]. One can envision multi‐site IS trials being embedded across these networks, as evidenced by the MEDIC ALERT PE study [40] conducted across 12 Michigan Emergency Departments (ED) to evaluate a four‐component implementation program aimed at improving home‐based treatment for low‐risk acute pulmonary embolism patients. Each participating ED is part of the Michigan Emergency Department Improvement Collaborative, which abstracts data from EHRs to empower clinical champions in driving quality improvements. By combining private resources with a public health focus, these partnerships may accelerate trial deployment and foster shared responsibility for healthcare improvement.

In addition to national collaborations, international partnerships are important for achieving a global impact in implementation science. Implementation challenges often cross borders, and solutions developed in one country may provide valuable insights for others. By participating in international collaborations, healthcare systems can share knowledge on best practices and adapt successful strategies to diverse settings. The HIV Prevention Trials Network (HPTN) is a pioneer in such global efforts [41]. It enables multiple countries to pool resources and expertise to develop strategies to reduce HIV in diverse populations. It exemplifies how international partnerships can enable the development of robust, widely applicable strategies capable of addressing complex health challenges globally.

The patient partnership is also essential for implementation trials to thrive. Patients and caregivers provide unique, lived insights into healthcare needs and strategy impacts, helping to shape trials that are meaningful and aligned with real patient needs. By engaging patients, families, and caregivers as active partners rather than passive participants, healthcare systems can improve trial design, enhance patient recruitment and retention, and increase the likelihood of meaningful outcomes. Partnerships with national patient advocacy organizations can support both the design and execution of IS trials across health systems. Transparent communication and respectful collaboration are needed to build trust and foster a sense of ownership, making implementation trials more relevant to the populations they aim to serve and more equitable in ensuring that diverse voices, particularly those from underserved groups, are included in shaping research and its outcomes.

### 3.4 Cultivate a New Culture

In current health systems, IS remains less widely adopted as a norm compared to QI despite their shared goals of improving health outcomes. Within health systems, there is a need to clarify the relationship between QI and IS, particularly in terms of terminology and scope. Educating practitioners in both fields is key to facilitating communication and collaboration [42, 43]. For instance, implementation trials and IS principles could be incorporated into medical and QI curricula, giving clinicians and quality leaders early exposure to these methods. Many researchers have now called for incorporating IS methods into improvement practice to better integrate evidence‐based interventions into routine care and to define generalizable methods that will accelerate systematic outcome improvement. These methods can help address the gaps in understanding the impact of QI strategies on healthcare delivery [44]. For example, implementation trials could rigorously evaluate the effectiveness of QI strategies, providing evidence that can inform and refine Plan‐Do‐Study‐Act (PDSA) cycles. In the meantime, IS addresses multi‐level factors and strategies that influence the success and sustainability of interventions. This includes understanding contextual barriers and facilitators, which are often overlooked in QI alone [42]. Cultivating a culture that values this integration is crucial for transforming improvement initiatives into systematic, scalable learning processes that accelerate progress toward population‐wide health goals.

## 4 Conclusion

Data‐driven implementation trials act as catalysts to optimize emerging learning health systems, supplying the rigorous comparative evidence needed to refine and scale implementation efforts. Embedding these trials into routine care accelerates iterative learning, addresses persistent barriers, and fine‐tunes best practices across diverse contexts. Although their potential is evident, the agenda remains aspirational even in established health systems such as the US and UK; however, the underlying principles offer a foundation that could also be adapted in smaller or resource‐limited settings. Realizing this promise will require further work to understand how such models can be tailored and sustained, but the direction of travel is clear. Now is the time for leaders, funders, and practitioners to invest in the infrastructure, partnerships, and culture needed to harness routine data and IS approaches for truly transformative healthcare.

## Conflicts of Interest

Dr. Beidas is the principal at Implementation Science & Practice LLC. She is currently an appointed member of the National Advisory Mental Health Council and serves on the scientific advisory board for AIM Youth Mental Health Foundation and the Klingenstein Third Generation Foundation. She has received consulting fees from United Behavioral Health and OptumLabs. She previously served as an appointed member of the NASEM study, “Blueprint for a national prevention infrastructure for behavioral health disorders,” and on the scientific and advisory board for Optum Behavioral Health. All activities are outside of the submitted work.

## Data Availability

Data sharing is not applicable to this article as no datasets were generated or analysed during the current study.
